# The creation, development and diffusion of the LARCG- latin american renal cancer group

**DOI:** 10.1590/S1677-5538.IBJU.2017.01.02

**Published:** 2017

**Authors:** Stênio de Cássio Zequi, Diego Abreu Clavijo, and all other LARCG, Members

**Affiliations:** 1 Urology Divison, AC Camargo Cancer Center, São Paulo, Brasil;; 2 Servicio de Urología, Hospital Pasteur Montevideo, Uruguay

## INTRODUCTION

As usually verified in many malignancies, the majority of the scientific information about renal cell carcinoma (RCC) is produced in developed countries mainly in North America and Europe. This knowledge is derived from great casuistries, joined in multi-institutional collaborative study groups or in International diseases consortiums.

Consistent epidemiologic and scientific data originated in the Latin America (LA) are lacking. LA is a large subcontinent, composed by more than 20 countries (much of them great economies), encompassing around 640 million habitants. Latin American population ethnicity is unique, due to an intense miscegenation, differing from northern hemispheric populations. The LA’s population was composed by several civilizations over the years: pre Colombians, (Amerindians), black slaves descendant’s (distinct groups of the African slaves that were sent to North America and Caribe). The predominance of Europeans in LA corresponded to Iberians, and Italians, few French and Germans. We have few Anglo-Saxon, Scandinavian and Northern and Eastern Europeans. Regarding Middle East and Africans, the more prevalent immigrants were Syrian, Lebaneses, Jewishs and few Armenians. Also, there are few Arabic, Persian and North African populations. From Asia, the predominance has been established by Japanese and in the last decades, by some Korean and Chinese. There is almost no people from South Asia, Oceania and Pacific Islands etc., differing from US, for example. The LA racial miscegenation resulted in particular genetic groups such as Mulattoes, Mestizos, Zambos, Cimarron’s, Cafuzzos, mamelucos etc (1, 2). In the era of molecular biology, and “omics”, it seems amazing to know the demographic, clinical, pathological and biomolecular profiles of several cancers in different populations. The research opportunities in these LA populations are exciting.

However, the socioeconomic level and the human development index among LA’s populations in general are under than desired. Health care systems in our subcontinent are heterogeneous, being possible to find in a same country (or in a same city), side by side, the more developed, and the more precarious medical services. Additionally, the Latin American Institutions, and its practitioners are not skilled in participating in multicenter collaborative study groups. The language may constitute an additional barrier, since the number of non-native English speakers (and writers) is large. At the same time, the background and the funds available to incentivize scientific productions are insufficient, in contrast with several primary and more urgent health requirements in LA.

### The LARCG

In face of this scenario, in May 2013, during the 108^th^ American Urological Society Annual Meeting in San Diego, colleagues from Brazil (SCZ) and Uruguay (DAC), decided to create an international, multicenter, nonprofit, collaborative study group, focused on kidney cancer, named LARCG (Latin American Renal Cancer Group).

The main generic role of LARCG is to promote knowledge and the research development regarding renal cell carcinoma (RCC) in LA. In order to reach this, it has become necessary to aggregate people and Institutions dedicated to this disease.

### Aims and consolidation

Among the scopes of LARCG, the main ones were: 1) the creation of a great and multifaceted data bank with information of RCC in LA; 2) to stablish international scientific cooperation between the LARCG institutions and developed research centers or with collaborative uro-oncological intergroups; 3) to proportionate facilities that result in the production of high level scientific publications; 4) To stimulate the participation of LARCG members in worldwide recognized scientific meetings.

The first step to concretize our aims was to invite LA’s key opinion leaders, to participate, and we had a quick and massive adhesion (special thanks to the work developed by Dr. Alejandro Nolazco, from Argentina in this task). On 2014, after a few months we had the adhesion of 24 institutions from six countries (Brazil, Uruguay, Argentina, Chile, Mexico and Spain), constituting the first round of the LARCG.

At the same time, through electronic assemblies, a statute was approved by all LARCG members. According to this agreement, it was created the Executive Directory, the Member’s Council, and the Scientific and the Ethics Committees. In each participating center, an Urologist Leader (UL) was nominated.

The UL must diffuse the LARCG ideas on each center recruiting urologists, and designating expert uro-pathologists, and clinical oncologists. These colleagues constitute the LARCG Pathol Branch and the LARCG Oncol Branch respectively, reinforcing the multidisciplinary characteristics of the group.

Today, LARCG is active, supported by annual fees paid by its members and is consolidated: www.larcg.org (3).

### Scientific Steps

The first step in the scientific direction was the creation of an extensive data bank, containing 176 demographical, clinical, laboratorial and pathological variables, all of them previously codified and with careful protection of the patients’ identifications. This data bank was sent to 24 institutions participants of the first round of the LARCG, in January 2014, and there were six months for the return of the information. At the end of 2014, we received information from 4280 RCC Patients. From these group, 3817 patients were eligible for a realization of the “*First epidemiological survey and an early survival analysis”*, form LARCG. These results were presented for the first time at the Main Session of the CAU - Confederacion Americana de Urologia Meeting, in Punta del Este Uruguay, in November, 2014. During this congress, it was realized the *First Annual LARCG Meeting*, with the presence of the majority of our members. Few months later, in 2015 April, these first analyses results, and the structural aspects of the LARCG, were presented at the “2015 Spring SWOG (Southwest Oncology Group) Meeting”, in San Francisco, California. (thanks to the friendship of the SWOG, in the names of Drs. Ian M. Thompson, MD, Primo N. Lara MD, and Manuel Valdivieso, MD). After a few weeks, LARCG performed it’s *Second Annual Meeting* during the 2015 AUA Annual Meeting in New Orleans LO, in a meeting room supported by the AUA (special thanks to the personal collaboration performed by Shlomo Raz, MD).

LARCG promoted Scientific International Meetings in São Paulo, in March 2015, at AC Camargo Cancer Center, and in October, 2015, in the British Hospital in Buenos Aires.

In the end of 2015, the first LARCG season of submission of scientific projects was opened, and we received several applications, fourteen of them were approved by the Ethics and the Scientific Committees. All of these projects were presented during the *Third LARCG Annual Meeting*, realized in San Diego, during the 2016 AUA Meeting, under the support of the AUA. In this Section, we were proud to receive a special honored Guest, W. Marston Linehan, MD.

During the 2016 Meeting of the Associacion Argentina de Urologia, in Tucuman-Argentina), “Contemporary outcomes in the Management of Metastatic Renal Cell Carcinoma from the Latin American Renal Cancer Group (LARCG)” was presented (4). In October 2016, at the SIU - Societé Internationnale D’ Urologie International Meeting in Buenos Aires, two abstracts were presented: one reporting the creation and development of the LARCG (5) and the other evaluating the Role of the American Society of Anesthesiology Classifications as a prognostic Factor in RCC (6).

The second Round: In 2015 LARCG finalized the adhesion of 45 centers, from Brazil, Uruguay, Argentina, Chile, Peru, Bolivia, Mexico and Spain. At this time, our Scientific Committee corrected and enhanced our data bank, and sent it to all centers, that had some months to fill it and/or to correct the previous imprecisions and return it to us. At the end of 2016, we finalized the second round, having information of 5223 patients (sent by 35 of our centers – there are 10 inactive centers, yet). The information is customized in a data bank. Now the specific variables’s informations has been sent to the LARCG investigators which had aprooved research projetcs.

### LARCG Pathologic Branch Activities

One of the most significant initiatives of the LARCG is to integrate the demographic, clinical data with pathological samples of each respective patient. For this task, we must acknowledge the non-interested collaboration of Dra. Isabela Werneck da Cunha, MD, PhD, (Leader of the LARCG Pathol), the LARCG Pathologists, and the personal contribution of Dr. George Netto, MD. Those skilled pathologists are centrally reviewing and reclassifying thousands of samples, according to the 2012, ISUP - International Society of Urological Pathology Consensus (7). From each case, samples of tissue microarray have been prepared (there were more than 750 samples of clear cell RCC from Brazil and Uruguay, already prepared). Argentinian Pathologists are reviewing their cases at this time. The junction of clinical, epidemiological data and pathological samples, for sure, will result in multiple opportunities for research and assays. In the future, biological fluids, frozen tissues etc. might be joined, under rigorous ethical and legal cares.

### International Collaborations

There is a signed international agreement between two LARCG Centers (AC Camargo Cancer Center, Brazil and Pasteur Hospital, Uruguay) and Lee Moffitt Cancer Center, from Tampa, US, through Dr. Philippe P. Spiess, MD and Jorge Lockhart, MD.

Other collaborations has been stablishing with one of the SWOG’s centers (University de California Davis-thanks to Dr. Primo Lara, MD) and AC Camargo Cancer Center. Some LARCG centers will collaborate with the recently aproveed project INCITO-INOTE (founded by Brazilian Offical fomment Agencies) 8, leadeared by VIlma Regina Martins, MSc, PhD.

### Plans for 2017 and beyond

For 2017, LARCG web site, originally in Spanish, will become bilingual, including the English idiom. Periodically, at our home page, newsletters, editorials, clinical cases discussions will be available.

The Next *LARCG Annual Meeting (4* ^*th*^ *)* will be held in Boston 2017, during the next AUA Annual Meeting. During this meeting, the research projects underway will be presented, and worldwide recognized RCC Speakers will be invited.

Negotiations are in course to establish future collaborations with international research groups and with RCC patient’s protection and educational groups. We are now looking for a sponsor institution, that can support LARCG in its several and infrastructural logistics requirements.

We wish a bright 2017 for all LARCG members, collaborators and of course, for our patients.

Stênio de Cássio Zequi, MD, MSc, PhD Editor Associado, International Braz J Urol Divisão de Urologia do A.C. Camargo Cancer Center Fundação A. Prudente, São Paulo, Brasil
